# A Case Report on Dyskinesia Following Rivastigmine Patch 13.3 mg/24 hours for Alzheimer's Disease: Perspective in the Movement Disorders Spectrum Following Use of Cholinesterase Inhibitors

**DOI:** 10.1097/MD.0000000000001364

**Published:** 2015-08-28

**Authors:** Maria Cristina B. Diaz, Raymond L. Rosales

**Affiliations:** From the Department of Neurology and Psychiatry, University of Santo Tomas Hospital, Espana, Manila, Philippines.

## Abstract

Current reports on movement disorder adverse effects of acetylcholinesterase inhibitors only include extrapyramidal symptoms and myoclonus.

Here is a case of an 81-year-old female Filipino with dementia who presented with first-onset generalized choreiform movements.

The etiology of the clinical finding of dyskinesia was investigated through laboratories, neuroimaging, and electroencephalogram, all of which yielded negative results. Review of her medications included the rivastigmine (Exelon) patch, which had just been increased to 13.3 mg/24-hour-dose 3 months prior. With all other possible causes excluded, a trial discontinuation of rivastigmine, showed decreased frequency of the dyskinesia 48 hours after, with complete resolution after 6 days, and no recurrence since then.

This case thus presents a probable association or causality between the choreiform movement and rivastigmine at 13.3 mg/24-hour-dose patch because of clear temporal proximity, lack of alternative explanations, and a reversal of the dyskinesia upon medicament discontinuation.

## INTRODUCTION

Majority of research comparing cholinesterase inhibitors show no significant difference in drug efficacy but instead in the incidence of adverse effects. In 2006, Lockhart et al^1^ performed a systemic review on the safety and tolerability of these acetylcholinesterase inhibitors. Apart from the common gastrointestinal symptoms, neurologic manifestations including headache, dizziness, vertigo, syncope, change in sensorium, confusion, irritability, agitation, hallucinations, seizure, and extrapyramidal symptoms may also occur as seen in 7 of the 12 studies reviewed.^2,3^ Only the study by Sobow and Kloszewska^2^ showed consequent motor dysfunction termed as extrapyramidal symptoms however its phenomenology was not described. These were seen in 2 patients under the donepezil (5–10 mg/day) group and 3 patients under the rivastigmine (6–12 mg/day) group.

This case report is documentation of an uncommon adverse event related to rivastigmine (Exelon) 13.3 mg/24-hour patch in the form of choreiform movement.

## PATIENT INFORMATION

This is a case of an 81-year-old female, Filipino, who presented with involuntary movements. Diagnosed with severe dementia of the Alzheimer's type, she is dependent in all activities of daily living, including mobility out of bed. She is able to respond to others with subtle facial expressions but has no comprehensible verbal output. She has hypertension, cardiac arrhythmia, coronary artery disease, and deep venous thrombosis. She has a cardiac pacemaker, which was placed due to sick sinus syndrome. Her maintenance medications included amiodarone, metoprolol, rivaroxaban, and patch rivastigmine. Quetiapine was sparingly administered at half to one tablet of 25 mg, as necessary for insomnia and restlessness.

In particular, the patient has been on rivastigmine transdermal patch since April 2009 at a dose of 4.6 mg/24 hours, and which was increased to 9.5 mg/24 hours in August 2009. In these doses of rivastigmine, no note of adverse events occurred. Because of the progression of her dementia symptoms, the rivastigmine dose was increased to 13.3 mg/24 hours in March 2014.

## CLINICAL FINDINGS, TIMELINE, AND DIAGNOSTIC ASSESSMENT

**FIGURE 1:**
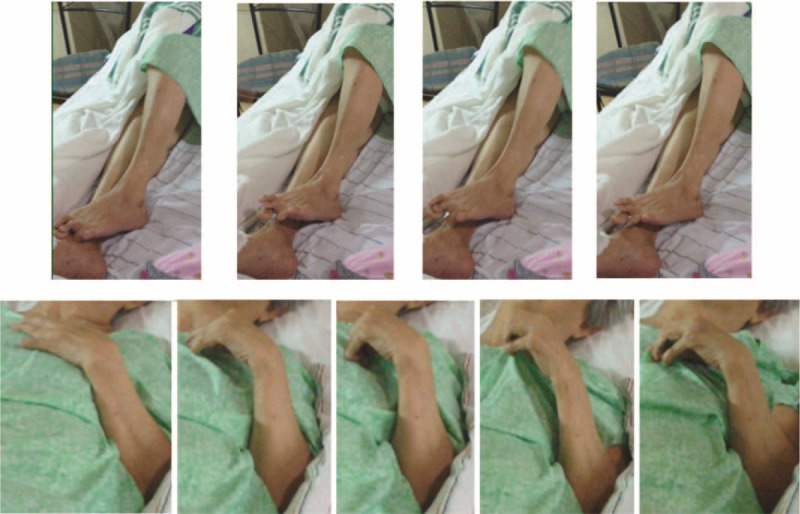
A series of still images demonstrating the observed choreiform movement.

The patient was apparently well until June 13, 2014, when she developed involuntary movements of the left lower extremity. This was described as fluid, jerky and at times flinging in character, with similar observations later involving her left upper extremity and the right extremities as well. She remained alert and responsive, and was immediately brought to their local hospital (see Figure 1).

**FIGURE 2:**
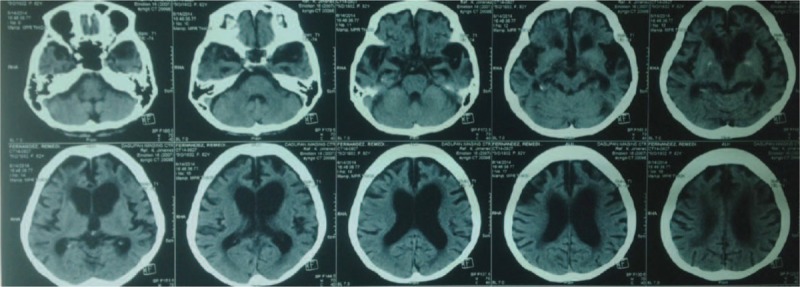
Cranial CT scan (June 15, 2014) with no evident lesions over the basal ganglia and brainstem.

With an initial clinical impression of a stroke, a plain cranial CT (computerized tomography) scan was done which showed no new noticeable lesions (see Figure 2). For lack of a possible metabolic cause, and seizure being entertained, an electroencephalogram (EEG) was also performed. Apart from a generalized slowing of background activity, no epileptiform discharges were found. The attending physician opted to empirically administer 1 dose of 250 mg levetiracetam tablet and 1 dose of 1 mg risperidone tablet, before transfer to our University hospital. During this time, the described abnormal movements neither abated, nor worsened.

Upon arrival at the university hospital, she was conscious and was able to regard, but with limited ability to follow commands. Her pupils were isocoric with intact direct and consensual light reflex. The gaze was midline, with conjugate eye movements. There were no evident cranial nerve deficits. There was no motor weakness; however, the choreiform movements described above were observed on all 4 extremities, more frequently seen on the left. There was bilateral Babinski.

To investigate the etiology of the observed choreiform movement, a cranial MRI would have been ideal; however, the presence of a cardiac pacemaker contraindicated it. A thorough review of the cranial CT scan showed no lesions over the basal ganglia and upper brainstem. The ensuing hematologic, biochemical, electrolyte, and thyroid function tests were not yielding. No other metabolic or infectious events seemed to have caused the choreiform movements. Her medications were reviewed, and the only newly introduced medication for the past 3 months was rivastigmine at a higher dose of 13.3 mg/24 hours/patch. Upon review, no treatment gaps occurred between rivastigmine dosages and the old patch was removed after 24 hours (ie, on the same hour daily) before applying the new one, while ensuring a daily change of patch application at recommended sites. Vomiting did not occur, and neither was the patient administered metoclopramide.

## THERAPEUTIC INTERVENTION

Levetiracetam was discontinued as the movement as described was not seen to be a form of seizure. A trial discontinuation of the transdermal patch was done on June 15, 2014. A marked reduction of frequency of the choreiform movement was seen 2 days after, and a complete resolution was seen after 6 days. An 1-time rechallenge with the same patch dose was tried, which again showed reemergence of the choreiform movements. Thus, rivastigmine 13.3 mg/24 hours/patch was discontinued, and the family was not keen on introducing the patch again, even at previous lower doses. Choreiform movements did not recur, up until this present report.

## DISCUSSION

The reversal of the choreiform movements after discontinuation of rivastigmine, and its recurrence on the same drug challenge, shows a possible causality worth investigating. Notably, rivastigmine was the only drug that had a dose augmentation, while the other medications on board were kept at maintained doses. Investigation of the etiology of the choreiform movement was as previously mentioned limited by the cardiac pacemaker not allowing for more sophisticated neuroimaging.

Amiodarone may inadvertently cause neurologic like tremors, parkinsonism, myoclonus, and various dyskinesias, which are linked to idiosyncratic hyperthyroidism.^4^ The patient who has been chronically maintained on amiodarone had no history of any dyskinesia. Trial discontinuation of the rivastigmine transdermal patch was followed by complete resolution of the symptom.

The complexity in interpreting the choreiform movements in this case lies in that the present case has been receiving occasional low-dose second-generation antipsychotic agent (SGA; quetiapine, half to one tablet of 25 mg/day, as necessary) since 2012. Movement disorders, whether it be acute dystonia, or be tardive dystonia/dyskinesia (choreiform movements included), are “extrapyramidal symptoms (EPS)” that commonly appear among the first-generation antipsychotic agents (FGA; eg, haloperidol), after an acute use (days to weeks) or after a chronic use (months to years). Such EPS are also reported in SGA's, but not as prevalent as one would anticipate in the FGA's.^5^ In the case of quetiapine, the EPS is expectedly less, as against the other SGA's, and appears late in the course of treatment (ie, commonly given at more than the doses applied in this present case), and usually occurs after the dosage is decreased or after the drug is discontinued. The dyskinesia often persists for months to years after the discontinuation. Its response to any type of therapy is usually poor.^6^

Although applied sparingly in this present case, the much safer quetiapine^7^ was being given at lower doses (12.5–25 mg/occasion), and thus EPS would not be an expected cause of the choreiform movements. A single dose of 1 mg risperidone given elsewhere did not also change the clinical occurrence of choreiform movements of this present case.

Cases of EPS are reportedly rare in a number of dementia syndromes given anticholinesterases. Donepezil was found to induce cervical dystonias (ie, antecollis^8^ and laterocollis,^9^ including myoclonus^10^) for cases with Alzheimer's disease, Parkinson's disease dementia, and Lewy-body dementia. “Pisa syndrome,” or axial deviation, also occurred in dementia cases given galantamine^11^ and donepezil.^9,12^

Rivastigmine-induced dystonia was reported in the past, both in the oral^13^ and patch^14^ formulations. In the latter case,^14^ the rivastigmine patch was commenced at 5 mg/cm^2^, with good tolerability, and a month later, the dose of rivastigmine patch was increased to 10 mg/cm^2^. Dystonia occurred in their case when rivastigmine patch was augmented, but which abated on discontinuation, and reemerged on the same dose patch application.^14^ It would seem that this present case of ours parallel the latter case, wherein, a dose augmentation to patch 15 mg rivastigmine led to the choreiform movements, but which abated on discontinuation, and reemerged on rechallenge of the same medication.

Dystonic reactions may occur because rivastigmine possesses not only a nonlinear pharmacokinetics, but that it also inhibits both acetylcholinesterase and butyrylcholinesterase enzymes, potentially leading to increased muscle tone. The excessive increase in cholinergic activity due to anticholinesterase action of rivastigmine in the brain may disrupt the usual balance between dopaminergic and cholinergic receptors in the basal ganglia.^12^ One may postulate that the concurrent medications may also reduce the threshold for development of dyskinetic reactions.^13,14^

Rivastigmine targets the G1 enzymatic form of acetylcholine mostly in the hippocampus and cortex of individuals with Alzheimer's disease. Although of similar chemical structure, unlike organophosphates, which bind irreversibly, and carbamates, which bind reversibly for 8 to 10 hours, rivastigmine binds acetylcholinesterase for only 4 to 6 hours.^15^ Similar to the treatment with acetylcholinesterase inhibitors, in organophosphate poisoning, the resultant increase in central cholinergic tone consequent to the imbalance between acetylcholine and dopamine in the substantia nigra and basal ganglia lead to EPS symptoms. Hsieh et al^16^ proposed that an abundance of acetylcholine, such as when its inhibitor is blocked, may impair basal ganglia function as a genetically determined critical low threshold of acetylcholine is necessary to regulate dopaminergic activity within the basal ganglia. According to Muller-Vahl et al,^17^ dysfunction in the cortico-striato-pallido-thalamo-cortical circuits is influenced by biochemical alterations leading to dopamine deficiency. Reports of choreoathetosis, opisthotonos, dyskinesia, dysarthria, dystonia (oral, focal, general), typical parkinsonism (masked fascies, bradykinesia, rigidity, tremors, shuffling gait), and EPS (facial grimacing, tongue protrusion, acute oculogyric crisis) are among the movement disorders linked to organophosphates.

This case reflects an adverse effect, which has not been seen documented in other studies. The reversal of choreiform movements after discontinuation of rivastigmine (13.3 mg/24-hour dose) poses a causal association that should be further looked into.

## INFORMED CONSENT

As the patient is no longer capable to give consent for reporting and publication of this case, her daughter as legal representative and nearest of kin consented in her behalf.
